# Drought and plant litter chemistry alter microbial gene expression and metabolite production

**DOI:** 10.1038/s41396-020-0683-6

**Published:** 2020-05-22

**Authors:** Ashish A. Malik, Tami Swenson, Claudia Weihe, Eric W. Morrison, Jennifer B. H. Martiny, Eoin L. Brodie, Trent R. Northen, Steven D. Allison

**Affiliations:** 1 Department of Ecology & Evolutionary Biology, University of California, Irvine, CA USA; 2 School of Biological Sciences, University of Aberdeen, Aberdeen, UK; 3 Environmental Genomics and Systems Biology Division, Lawrence Berkeley National Laboratory, Berkeley, CA USA; 4 Department of Earth System Science, University of California, Irvine, CA USA; 5 Earth and Environmental Sciences, Lawrence Berkeley National Laboratory, Berkeley, CA USA; 6 Department of Environmental Science, Policy and Management, University of California, Berkeley, CA USA

**Keywords:** Microbial ecology, Biogeochemistry

## Abstract

Drought represents a significant stress to microorganisms and is known to reduce microbial activity and organic matter decomposition in Mediterranean ecosystems. However, we lack a detailed understanding of the drought stress response of microbial decomposers. Here we present metatranscriptomic and metabolomic data on the physiological response of in situ microbial communities on plant litter to long-term drought in Californian grass and shrub ecosystems. We hypothesised that drought causes greater microbial allocation to stress tolerance relative to growth pathways. In grass litter, communities from the decade-long ambient and reduced precipitation treatments had distinct taxonomic and functional profiles. The most discernable physiological signatures of drought were production or uptake of compatible solutes to maintain cellular osmotic balance, and synthesis of capsular and extracellular polymeric substances as a mechanism to retain water. The results show a clear functional response to drought in grass litter communities with greater allocation to survival relative to growth that could affect decomposition under drought. In contrast, communities on chemically more diverse and complex shrub litter had smaller physiological differences in response to long-term drought but higher investment in resource acquisition traits across precipitation treatments, suggesting that the functional response to drought is constrained by substrate quality. Our findings suggest, for the first time in a field setting, a trade off between microbial drought stress tolerance, resource acquisition and growth traits in plant litter microbial communities.

## Introduction

Drought is common in terrestrial ecosystems, and climate change is making drought more frequent and severe [1, 2]. Mediterranean ecosystems like those in California, USA, that experience summer drought are particularly vulnerable to climate change through reduced winter precipitation and increased evapotranspiration that intensifies drought effects [3]. Drought affects biogeochemical processes through mechanisms including limitations to resource diffusion and transport as well as organismal physiological responses to water stress [4–7]. Both mechanisms may cause a decline in growth and activity of microbial decomposers [6, 8]. As a direct effect of water limitation, microorganisms use their cellular machinery to maintain osmotic balance with the surrounding environment which involves intracellular accumulation of solutes or altering of the cell envelope to retain water [5, 6, 9, 10]. Water limitation also affects microbial growth and survival indirectly by altering substrate transport and cellular motility [6]. Selection based on these physiological adaptations influences microbial community composition through changes in taxa abundance and genetic variation [6, 11, 12]. Still, we do not have a thorough understanding of the key physiological adaptations of in situ microbes to long-term drought. This knowledge gap introduces uncertainty in predictions of ecosystem processes under environmental change.

Long-term drought selects for stress tolerant microorganisms, and it also alters plant communities [13] and therefore plant litter chemistry that in turn can select for different microbial communities in the litter layer and the soil below [7, 12, 14, 15]. Selection based on the chemical quality of litter substrates could affect community physiology related to resource acquisition (substrate discovery, breakdown and uptake) and will likely impact drought tolerance and overall fitness of populations in the community [12, 16]. Thus, the indirect effects of drought via changes in plant litter chemistry could modify collective community physiology and therefore ecosystem process rates [7, 12]. An assessment of drought impact on microbial physiology and its biogeochemical implications must therefore also consider these indirect effects.

The overall aim of this study was to identify the physiological responses to long-term drought in microbial communities on decomposing plant leaf litter—the surface layer of soil. We investigated the stress physiology of distinct litter communities in grassland and shrubland ecosystems undergoing field precipitation manipulation for a decade, with the drought treatment involving a ~40% reduction in annual precipitation that results in reduced decomposition rates [7]. The metabolism of the active microbial community was assessed using metatranscriptomics (community gene expression) and metabolomics (endo and exometabolites) to analyse community functional shifts in response to chronic drought stress. We hypothesised that exposure to 10 years of drought has led to greater microbial allocation to stress tolerance relative to growth pathways. In the absence of chronic stressful conditions like drought, microbial communities should divert fewer resources into stress tolerance and therefore more into growth. Specifically, we hypothesised that (1) long-term drought causes increased gene expression and metabolite production associated with osmoprotection, dormancy and moisture retention mechanisms which leads to reduced growth; and (2) chemically diverse and complex shrub litter requires increased investment in resource acquisition pathways, further constraining microbial growth under drought. Our results demonstrate multiple physiological signatures of drought stress and resource limitation in decomposing litter microbial communities.

## Methods

### Field site description

The study site is part of the Loma Ridge Global Change Experiment situated near Irvine, California, USA (33°44′N, 117°42′E, 365 m elevation). The climate is Mediterranean with mean annual temperature of 17 °C and mean annual precipitation of 325 mm. Most precipitation falls between November and April, stimulating plant growth, and a summer drought lasts from May to October. The vegetation at the site includes annual grassland adjacent to coastal sage shrubland which correspond to the two litter types used in the experiment [7, 13]. The grassland plots (6.7 × 9.3 m) consisted of exotic annual grasses such as *Avena*, *Bromus*, and *Lolium* and forbs such as *Erodium*, whereas the shrub plots (18.3 × 12.2 m) consisted of crown-sprouting shrub species such as *Salvia mellifera*, *Artemisia californica* and *Malosma laurina*. This shrub litter is known to have higher *C*:*N* ratio, higher proportion of lignin and other recalcitrant compounds and lower proportion of cellulose, hemicellulose and cell solubles than the grass litter [17]. For our experiment, we used a subset of plots with field precipitation manipulations that have been established since February 2007. Reduced precipitation treatment involved ~40% reduction in precipitation compared with ambient which was achieved by covering plots with clear polyethylene during a subset of rain events each winter. The design was replicated to have four plots per treatment (grassland ambient, grassland reduced, shrubland ambient and shrubland reduced) for a total of 16 plots.

### Experimental design and sampling

Litter was collected in summer on 30 August 2017 from all four replicated plots within a treatment and homogenized by hand-mixing. Note that litter from each treatment was kept separate. Litter consisted of freshly fallen dry leaves in shrubland and dry senescing sheath and blade in grassland from leaf fall or grass senescence earlier in the year. Intact shrub leaf litter was used, whereas grass sheath and blade were cut to a length of ~10 cm. Litter bags were made by placing 6 g dry litter mass into 15 cm × 15 cm bags made from 1 mm mesh window screen. Bags were deployed on 12 September 2017. They were placed on top of the soil surface under the canopy. It was a particularly dry season and no substantial precipitation fell until December 2017. An in situ pulse wetting was performed on 30 November 2017 to simulate a discrete rainstorm of ~13 mm using previously collected rainwater (Fig. S1). Litter bags were collected before wetting (Sampling 1) and another batch of bags was collected the following day (Sampling 2). There was no precipitation in the following days and therefore the litter dried to pre-wetting moisture levels (Fig. S1). We collected another batch 12 days after wetting (Sampling 3). The in situ wetting treatment was either too small or too short to elicit a wet-up response (Figs. S2 and S3) [18]; hence we chose not to focus on the effect of wet-up and dry-down on microbial physiology but instead use all three sampling points to study the impact of vegetation type and long-term precipitation treatment on microbial community composition and physiology [12]. Sixteen litter bags were sampled at each time point (two vegetation types × two precipitation treatments × four replicates). Retrieved litter bags were immediately transported to the lab at room temperature. A subsample of leaf litter was ground in a mixer (a quick whirl for 5 s) and used for RNA and metabolite extraction.

### RNA extraction and sequencing

We carried out RNA extraction on a coarse-ground litter aliquot of 0.2 g for shrub and 0.5 g for grass using RNeasy PowerSoil Total RNA Kit following manufacturer instructions (Qiagen, Hilden, Germany). Due to a high amount of organic compounds co-extracted from shrub litter, a lower amount of starting material was used in the extraction protocol to increase the RNA yield. After resuspending the RNA pellet in solution SR7 in the final step, purity and concentration of total RNA was assessed using a Bioanalyzer 2100 (Agilent, Santa Clara, CA, USA), Qubit fluorometer (LifeTechnologies, Carlsbad, CA, USA) and Nanodrop 2000 Spectrophotometer (Thermo Scientific, USA). Bioanalyzer-derived abundances of 18S and 16S rRNA in total RNA extracts were used to calculate fungal:bacterial (*F*:*B*) ratios. 200–500 ng of total RNA was used for subsequent steps. Ribosomal RNA was removed using a Ribo-Zero rRNA Removal Kit (Illumina, San Diego, CA, USA) according to the manufacturer’s instructions with a modification that included combining magnetic beads from the yeast and bacteria kit as follows: 0.5× Yeast Removal Solution, 0.25× Gram Negative Bacteria Removal solution, and 0.25× Gram Positive Bacteria Removal Solution. Strand-specific and barcode indexed RNA-seq libraries were then generated using the Kapa RNA-seq Hyper kit (Kapa Biosystems, Cape Town, South Africa) following the instructions of the manufacturer. The fragment size distribution of the libraries was verified via micro-capillary gel electrophoresis on a Bioanalyzer 2100. The libraries were quantified by fluorometry on a Qubit fluorometer and pooled in equimolar ratios. The pool was quantified by qPCR with a Kapa Library Quant kit (Kapa Biosystems) and sequenced on one lane of an Illumina HiSeq 4000 (Illumina, San Diego, CA, USA) with single-end 100 bp reads. The sequencing was carried out at the DNA Technologies and Expression Analysis Cores at the UC Davis Genome Center.

Resulting sequences from metatranscriptomic analysis were annotated with the Metagenomics Rapid Annotation using Subsystems Technology (MG-RAST) server version 4.0.3 [19]. Functional classification was performed using the SEED Subsystems database and taxonomic annotations up to genus level were performed using the RefSeq database with a maximum *e* value cut-off of 10^−5^, minimum identity cut-off of 60% and minimum length of sequence alignment of 15 nucleotides. Abundance tables derived from MG-RAST (Data S1) were imported into R for downstream analyses. Some samples were excluded from further analyses as we either could not extract good quality RNA (extraction from shrub litter was difficult) or the quality of sequences obtained was poor (due to poor quality RNA or bad sequencing runs). Number of replicates (*n*) for each treatment combination was: grass ambient sampling 1: 4, grass ambient sampling 2: 3, grass ambient sampling 3: 4, grass reduced sampling 1–3: 4, shrub ambient sampling 1: 4, shrub ambient sampling 2: 2, shrub ambient sampling 3: 3, shrub reduced sampling 1: 4, shrub reduced sampling 2: 2, and shrub reduced sampling 3: 3.

### Metabolite extraction and analysis

Coarse-ground leaf litter samples (1 g) were placed in 50 mL tubes with the addition of LC–MS grade water (20 mL, Honeywell Burdick & Jackson, Morristown, NJ, USA). Two extraction controls were included at this step by adding 20 mL of water to empty tubes. All samples were extracted for 1 h on an orbital shaker (Orbital-Genie, Scientific Industries, Bohemia, NY, USA) at 200 rpm at 4 °C followed by centrifugation for 15 min at 3220 × *g* at 4 °C. Supernatants (8 mL) were filtered through 0.45 um syringe filters (Pall Acrodisc Supor membranes) into 15 mL tubes, frozen then lyophilized. Dried extracts were resuspended in 1 mL LC–MS grade methanol (Honeywell Burdick & Jackson, Morristown, NJ, USA) on ice, vortexed for 10 s, sonicated for 10 min in an ice bath and placed at 4 °C overnight. Samples were centrifuged for 15 min at 3220 × *g* at 10 °C and further cooled at −20 °C for 10 min. Supernatants (850 µL) were transferred to 1.5 mL Eppendorf tubes and dried down with a Savant SpeedVac SPD111V (Thermo Scientific, Waltham, WA, USA) for 1 h with a final resuspension in ice-cold methanol containing internal standards (200 µL) and filtered through 0.22 µm centrifugal membranes (Nanosep MF, Pall Corporation, Port Washington, NY, USA) by centrifuging at 10,000 × *g* for 5 min. Samples (50 µL) were transferred to LC–MS vials for metabolomics analysis.

Extracts were analyzed using normal-phase LC–MS using a HILIC-Z column (150 mm × 2.1 mm, 2.7 μm, 120 Å, Agilent Technologies, Santa Clara, CA, USA) on an Agilent 1290 series UHPLC. The two mobile phases for metabolite separation were 5 mM ammonium acetate in 0.2% acetic acid (A) and 95% acetonitrile with 5 mM ammonium acetate in 0.2% acetic acid (B) at a flow rate of 0.45 mL/min with the following gradient: 100% B for 1 min, decreased to 89% by 11 min, down to 70% by 15.75 min and 20% by 16.25 min, held until 18.5 min then back to 100% B by 18.6 min for a total runtime of 22.5 min. The column temperature was maintained at 40 °C. MS data were collected on a Thermo Q Exactive (Thermo Fisher Scientific, Waltham, MA, USA) and MS–MS data were collected using collision energies of 10–40 eV. Metabolomics data were analyzed using Metabolite Atlas [20] with in-house Python scripts to obtain extracted ion chromatograms and peak heights for each metabolite (Data S2). Metabolite identifications were verified with authentic chemical standards and validated based on three metrics (accurate mass <15 ppm, retention time within 1 min, and MS/MS fragment matching). Data from internal standards and quality control samples (included throughout the run) were analyzed to ensure consistent peak heights and retention times. One sample from grass reduced precipitation treatment was lost during the extraction process, otherwise *n* = 4 for each treatment combination.

### Ordination analysis and diversity indices

Rarefaction, ordination and diversity analyses of metatranscriptomics-derived functions and taxonomic units as well as metabolites were performed using the *vegan* package [21], and NMDS plots were generated using the *ggplot2* package [22] under the R software environment 3.4.2 [23]. Treatment effects were estimated using permutational multivariate analysis of variance (PERMANOVA) based on Bray–Curtis dissimilarity distance between samples with the R-vegan function adonis. Shannon diversity index was calculated with rarefied functions and taxonomic units. Two-way factorial ANOVA and post hoc Tukey honest significant differences tests were performed to ascertain the effect of treatments on functional and taxonomic α diversity.

### Treatment effect size and indicator analysis

We used samples from three time points to measure the impact of vegetation type and long-term field precipitation manipulation on community physiology. The effect of reduced precipitation on the abundances of metatranscriptomics-derived functions at the upper level of SEED Subsystems classification was measured as the ratio of sum-normalised transcript abundance in level 1 classes in reduced and ambient precipitation treatments. One-way ANOVA was used to ascertain if this effect was significant. We did not perform *p* value correction for multiple comparisons and kept the interpretation of shifts at level 1 classes to the minimum. To identify those transcripts that were significantly enriched in either reduced or ambient precipitation treatments in the two litter types, we used pairwise indicator species analysis as implemented within the R library *labdsv* [24]. The indval score for each transcript is the product of the relative frequency and relative average abundance within each treatment, and significance (*p*) was calculated through random reassignment of groups (1000 permutations). This indval score ranges from 0 to 1; maximum score of 1 for a transcript denotes that it is observed in all samples of only one treatment group. Transcripts above a threshold indval score of 0.65 and with a *p* value smaller than 0.05 were considered significant (Data S3). Indval score was chosen to have enough stringency while also aiming to obtain a manageable and meaningful number of functional indicators. To identify vegetation-specific transcript indicators, we performed the indicator analysis on transcripts from ambient grass and ambient shrub treatments. We observed thousands of grass and shrub specific indicators. The threshold indval score for significance was raised to 0.7 for grass–shrub pairwise comparison to reduce the number of vegetation-specific indicators (Data S4). The frequency of indicator transcripts in level 1 functional classes was obtained using the R *plyr* package [25].

### Heatmap visualisations

Metabolite abundances were visualised using the heatmap function in R. Metabolites that were significantly higher in either ambient or reduced precipitation treatments across both litter types were identified using pairwise indicator species analysis as described above. Here, we did not separate samples from grass and shrub litter in order to reduce choosing litter-specific plant-derived metabolites as indicators. Metabolites above a threshold indval score of 0.6 and with a *p* value smaller than 0.05 were considered significant. A lower indval score was used here because a higher stringency gave very few indicators.

### Correlation analysis and visualisation

Correlations between metabolite abundances were performed to quantify the identified trade offs between traits related to growth and stress tolerance. We tested for negative bivariate correlations between significantly enriched metabolites in either reduced or ambient precipitation treatments across both litter types. Correlation matrix visualization was done using *ggcorrplot* package in R [26], separating samples from grass and shrub litter. Pearson correlation coefficient was used as a measure of the linear dependence between metabolites. From this correlation analysis, we chose key relevant metabolites that represent the clusters of significantly enriched metabolites to further visualize the metabolic trade offs across and between treatments. These key metabolites were aspartic acid and adenosine (growth indicators), and ectoine and 5-oxo-proline (stress indicators). The *psych* package in R [27] was used to obtain bivariate scatter plots with linear model fits, correlation coefficients (*r*) and *p* values denoting the strength of relationships among the representative metabolites, separately for samples from grass and shrub litter.

## Results

### Community functional and taxonomic diversity

**Fig. 1:**
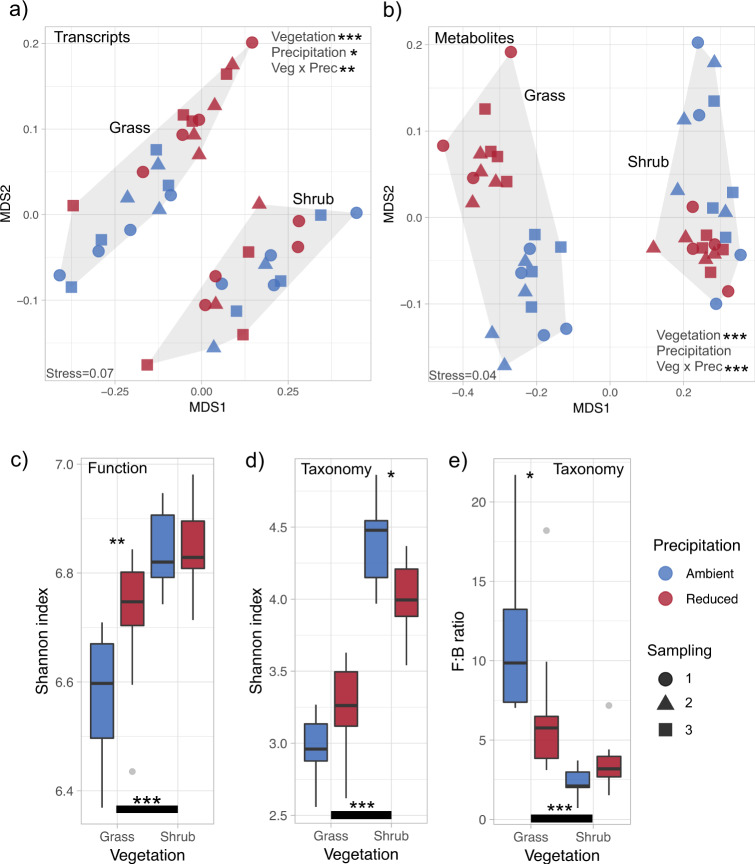
Community functional and taxonomic diversity. Two dimensional NMDS ordination of (**a**) transcripts (function) and (**b**) metabolites. *α* diversity of (**c**) functions and (**d**) taxa derived from transcripts across vegetation and long-term precipitation treatments; *n* = 9–12. Community fungal:bacterial (*F*:*B*) ratio (**e**) estimated as the ratio of mRNA sequences assigned to fungi and bacteria; *n* = 9–12. **a**, **b** Asterisks mark the significance of treatments that cause clustering of similar samples based on Bray–Curtis dissimilarity index analysed using permutational multivariate analysis of variance (PERMANOVA), and (**c**–**e**) asterisks mark the significance of differences between groups as analysed by Tukey’s multiple comparison test (***p < 0.001; **p < 0.01; *p < 0.05). Sampling time did not significantly influence any parameter.

Multivariate ordination analysis of transcripts (at the level of function in Subsystems classification) and metabolites suggested that the functional composition of communities did not differ across the three sampling points (Fig. 1a, b; PERMANOVA *p* > 0.05). Results across the sampling points indicated that grass and shrub litter communities were functionally dissimilar (Fig. 1a, b). Shrub litter communities demonstrated higher transcript functional *α* diversity than grass communities (Fig. 1c). In shrub litter, communities from reduced and ambient precipitation treatments shared more transcripts and metabolites than in grass communities where reduced precipitation clearly altered microbial physiology (Fig. 1a, b). Transcript functional α diversity was similar in shrub communities from ambient and reduced precipitation treatments (Fig. 1c). However, we observed a higher functional diversity of transcripts in grass communities from reduced precipitation treatment in comparison to ambient (Fig. 1c).

We used the taxonomic annotations of functional genes obtained from metatranscriptomics to ascertain the taxonomic α diversity and composition of the active litter microbial communities. Bacteria and fungi comprised ~97% of sequence reads. Communities from shrub vegetation had significantly higher taxonomic α diversity of transcripts than grass communities (Fig. 1d). Within each vegetation type, communities from ambient and reduced precipitation treatments differed taxonomically (Figs. S4 and S5), demonstrating a clear effect of long-term drought in shaping the taxonomic composition of the microbial community. Taxonomic composition of communities did not differ across the sampling points (Fig. S4). Fungi made up a large proportion of active communities in both grass and shrub vegetation. Fungal:bacterial ratios obtained from transcript annotations as well as from ribosomal RNA concentrations (Bioanalyzer-derived 18S:16S rRNA ratio) were higher in grass communities (Figs. 1e and S6). Relative abundance of fungi in communities was higher in grass litter under ambient precipitation compared with reduced precipitation (Fig. 1e). In shrub communities, there was no discernible shift in fungal:bacterial ratio in response to long-term precipitation treatment (Fig. 1e).

### Community functions unique to litter types

**Fig. 2:**
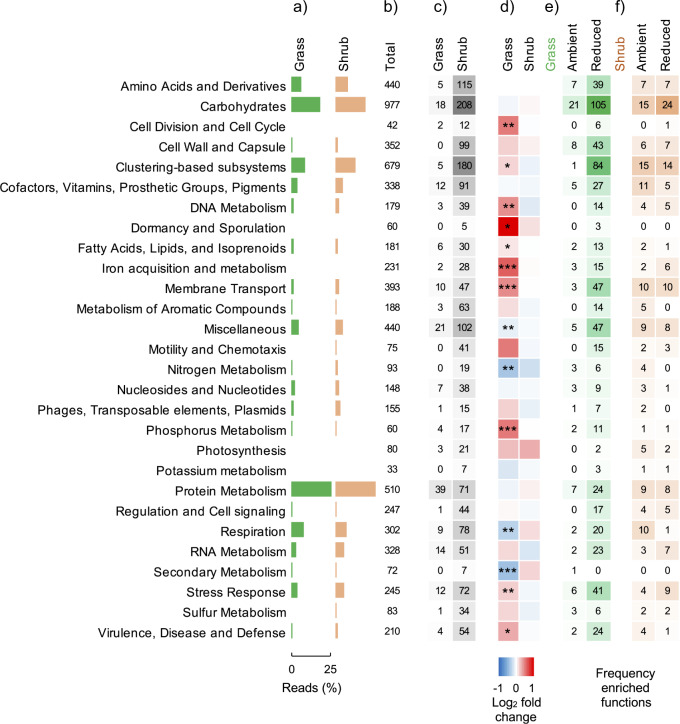
Impact of litter quality and long-term drought on microbial physiology. **a** Mean relative abundance of transcripts at level 1 of Subsystems classification in grass and shrub litter communities under ambient precipitation (*n* = 9–12). **b** Total number of genes annotated within each level 1 category (**c**) Frequency of significant transcript indicators (*p* < 0.05) in level 1 functional categories across litter type under ambient precipitation (*n* = 9–12). **d** Drought impact demonstrated as fold change in gene expression (at level 1) in communities from reduced precipitation relative to ambient. Asterisks mark significant drought-induced shifts in functional groups as analysed by one-way ANOVA (***p < 0.001; **p < 0.01; *p < 0.05; *n* = 9–12). **e**, **f** Frequency of significant transcript indicators (*p* < 0.05) in level 1 functional categories across precipitation treatments in (**e**) grass and (**f**) shrub communities (*n* = 9–12).

We used community-aggregated traits (means of functional traits found in a given community [28]) as a proxy to assess collective community physiology and its potential implications for ecosystem functioning. To identify traits that were unique to or significantly enriched in communities from either grass or shrub litter, we compared gene expression in communities from the two litter types under ambient precipitation treatment. The overall proportions of functions identified from metatranscriptomics were largely similar in communities from the two litter types (Fig. 2a). In total, 7141 Subsystems functions were annotated (Fig. 2b). We observed nearly ten times the number of unique functions in shrub communities compared with grass communities using indicator analyses (Fig. 2c, Data S4). Of the 182 functional indicators of grass communities, most belonged to the class protein metabolism (39 functions) and were linked to small and large subunit ribosomal protein synthesis as well as proteasome-mediated degradation of unneeded or damaged proteins [29]. On the contrary, we identified 1588 unique functions in shrub communities (Fig. 2c). The majority of functions unique to or enriched in shrub litter communities belonged to the class of carbohydrates (208 functions) and ranged from central carbohydrate metabolism to metabolism of mono-, di- and oligosaccharides, organic acids and sugar alcohols. In addition, 115 indicators of shrub litter communities were annotated to amino acid metabolism. The presence of a large number of functional indicators for carbohydrate and amino acid metabolism indicates increased investment in substrate degradation, uptake and assimilation in shrub communities. Microbial communities in shrub litter may have increased investment in these resource acquisition traits to degrade the more chemically complex and diverse substrates.

### Impact of long-term drought on gene expression

To uncover the physiological mechanisms that underlie microbial response to long-term drought, we analysed variable patterns in gene expression across the decade-long simulated precipitation treatments within each litter type. In grass communities, reduced precipitation caused systematic shifts in many functional classes (Fig. 2d). Functional classes that were significantly higher in relative abundance under reduced precipitation treatment were membrane transport, iron acquisition and metabolism, phosphorus metabolism, stress response, DNA metabolism, and cell division and cell cycle. Classes with significantly lower relative abundance under reduced precipitation treatment compared with ambient were secondary metabolism, respiration and nitrogen metabolism. These shifts in functional classes imply higher expression of conventional housekeeping genes in grassland ambient than reduced precipitation treatment. However, in shrub communities there were only small, non-significant changes in abundances of these functional classes in response to the 10-year reduced precipitation treatment (Fig. 2d).

Drawing conclusions from shifts in upper-level classes of functions may be misleading because this level consists of myriad genes whose expression may decrease or increase in response to the environmental perturbation [30]. Multiple genes belonging to the same class responding in opposite directions may cancel out resulting in no net effect for that class of functions. Therefore, we also assessed shifts in expression of individual genes to identify those that were unique to or significantly enriched in communities from either ambient or reduced precipitation treatments. From the 7141 Subsystems functions that were annotated in our dataset, in grassland communities we identified 665 transcript indicators of reduced precipitation treatment but only 87 indicators of ambient treatment (Fig. 2e, Data S3). In shrub litter communities, we observed similar numbers of indicator transcripts in contrasting precipitation treatments, with 140 under ambient precipitation and 120 under reduced precipitation (Fig. 2f, Data S3).

The majority of transcript indicators of reduced precipitation treatment in grass litter communities could be directly or indirectly linked to physiological adaptations to moisture stress. In contrast, most of the indicators of ambient treatment (Fig. 2e) belonged to common housekeeping functions (level 1 class carbohydrates: 21 functions). The highest number of transcript indicators of reduced precipitation in grass litter also belonged to the level 1 class of carbohydrates (Fig. 2e, 105 functions) but represented functions linked to metabolism of mono-, di- and oligosaccharides such as L-rhamnose, trehalose, maltose and maltodextrin, D-galacturonate, etc., and organic acids such as malonate. While some of these can be linked to conventional or alternative housekeeping functions, increased expression of genes linked to metabolism of sugars like trehalose suggests an adaptive mechanism of compatible solute accumulation to maintain cellular osmotic balance [5, 10, 31, 32].

A significant number of indicators of reduced precipitation in grass litter also belonged to the classes of membrane transport (47 functions) or stress response (41 functions) which were almost absent in the indicator profiles of ambient communities (Fig. 2e). Membrane transport functions were mostly annotated to multi-subunit cation antiporters (Na+ H+ antiporters), Ton and Tol transport systems, ABC transporters and protein secretion systems. Increased expression of genes for cation antiporters indicates a drought stress tolerance strategy of accumulating inorganic ions aimed at maintenance of cellular osmolarity [6, 9, 33]. In the level 1 class of stress response, we observed increased expression of genes related to uptake/biosynthesis of choline, betaine and ectoine. These metabolites are widely-reported osmolytes in plants and microorganisms [6, 9, 10, 31, 34] and provide additional evidence for osmolyte accumulation as a stress tolerance mechanism in drought communities.

**Fig. 3:**
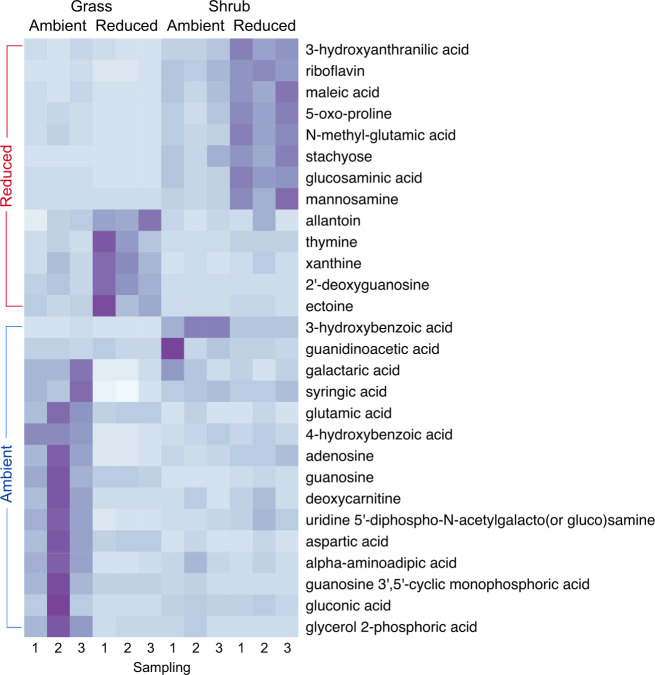
Metabolite abundance across litter type and precipitation treatments. Heatmap showing mean peak heights (*n* = 3–4) which relates to the abundance of metabolites that were significantly higher (*p* < 0.05) in either ambient or reduced precipitation treatments across both litter types. Rows of metabolites are clustered vertically by level of enrichment in either ambient or reduced precipitation.

We also observed enrichment of transcripts belonging to the cell wall and capsule class in communities from reduced precipitation in grass litter (43 functions, Fig. 2e). Significant gene indicators here were linked to metabolism of capsular and extracellular polysaccharides (EPS), Gram-negative cell wall components (lipopolysaccharide assembly), and peptidoglycan biosynthesis demonstrating strategies either to retain water through capsular or EPS “sponges”, or to lower the permeability of cell walls to avoid water loss [6, 9, 32]. Genes of the classes RNA and protein metabolism that were significantly higher in reduced precipitation treatments were related to functions like transcription, RNA processing, and protein synthesis/modification and were mostly of bacterial origin, whereas similar transcript indicators in ambient communities were mostly eukaryotic. This evidence links these functional indicators to drought-induced shifts in fungal:bacterial ratios of microbial communities (Fig. 1e) [7]. A high number of functional indicators belonged to clustering-based subsystems (84 functions, Fig. 3c) and miscellaneous (47 functions), but the majority of these were unassigned or putative functions that did not provide any clear functional insight.

Shrub litter communities were functionally more diverse than grass litter communities but precipitation treatment did not change the functional diversity (Fig. 1a–c). Correspondingly, we observed a similar number of unique functions across the contrasting precipitation treatments within shrub litter (Fig. 2f). Unlike grass communities, very few distinctive stress response functions were identified in shrub communities under reduced precipitation treatment; these were linked to trehalose metabolism, oxidative stress and multi-subunit cation (Na+ H+) antiporters. Thus, the drought stress response physiology of microbial communities is variable and could depend on other traits like resource acquisition linked to litter chemistry.

### Long-term drought impact on community metabolite production

We next identified metabolites that were unique to or observed in higher abundance in either reduced or ambient precipitation treatments (Figs. 3 and S7). The results corroborated the patterns revealed using metatranscriptomics. Most of the metabolites identified as indicators of precipitation treatment were monomeric compounds such as amino sugars, amino acids, ribonucleosides, and organic acids that are likely derived from living microorganisms. Although plants also produce many of these compounds, plant-derived monomers would likely have been metabolized by litter microbes prior to our measurements [35, 36]. Ectoine, which functions as a compatible solute to maintain internal water potential, showed greater abundance in the reduced precipitation treatment particularly in grass litter communities. 5-oxo-proline, choline and betaine are also potential osmolytes; these showed greater abundance in shrub litter communities under reduced precipitation treatment. On the contrary, in the grass litter under ambient precipitation treatment, various biogenic amino acids such as aspartic acid, and purine ribonucleosides such as adenosine and guanosine were enriched; these are molecular building-blocks of proteins and RNA, respectively and indicate growth and biosynthesis. The abundance of these growth indicators was higher in the wet litter samples (Sampling 2) relative to the drier (Sampling 1 and 3) indicating increased activity under higher moisture content, while the abundance of stress indicators like ectoine and 5-oxo-proline was higher in the dry samples (Figs. 3 and S2). This time-resolved pattern suggests that these metabolites are likely of microbial origin [18]. The differential metabolite profiles in contrasting precipitation treatments in grass communities corroborate the metabolic trade off between growth and water stress adaptations suggested by the transcriptomics data.

**Fig. 4:**
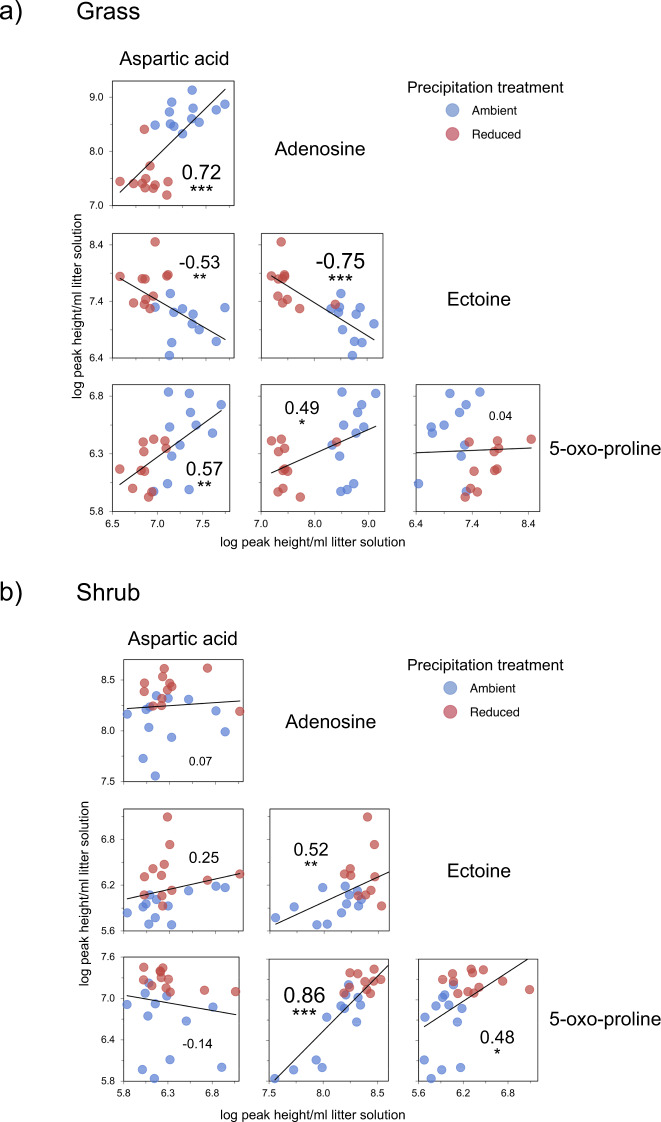
Distribution of metabolite abundance demonstrates metabolic trade offs. Linear regression trends of representative growth indicators aspartic acid and adenosine, and stress indicators (osmolytes) ectoine and 5-oxo-proline in (**a**) grass and (**b**) shrub litter communities. These metabolites were the most relevant from among those that were significantly enriched in either ambient or reduced precipitation treatments (shown in Fig. 3). Numbers within each scatter plot are correlation coefficients (*r*) and asterisks denote the significance of the relationship across treatments (***p < 0.001; **p < 0.01; *p < 0.05; *n* = 23–24).

Consistent with metabolic trade offs, we observed 47 and 27 significant negative correlations between metabolite pairs (out of 378 tested) in grass and shrub litter communities, respectively (Fig. S8). In grass litter communities, we observed significant negative correlations between relevant growth indicators like aspartic acid and adenosine and drought stress indicators like ectoine (Fig. 4). In shrub litter communities, although we did detect slightly higher amount of osmolytes ectoine and 5-oxo-proline in the reduced precipitation treatment, there were no negative correlations between these drought stress indicators and the growth indicators (Fig. 4).

## Discussion

We found distinctive physiological signatures of microbial communities growing on plant leaf litter in response to simulated decade-long drought. Given that natural rainfall was not observed or manipulated during our 2.5-month experiment, the differing gene expression and metabolite patterns across long-term precipitation treatments likely arose due to indirect legacy effects on microbial communities or litter chemistry driven by changes in plant communities [7, 12]. Among these possibilities, the physiological differences more likely result from drought-induced changes in microbial community composition and function [7, 37]. Community shifts also suggest that environmental pressures selected for taxa that can survive the physiological stress imposed by drought [38–41]. Although drought-induced changes in plant litter chemistry may also cause physiological shifts in the microbial community [7, 12], these differences were small enough (unpublished data) that they did not affect litter decomposition rates as demonstrated in a previous study at the same site [7].

For the first time in a field setting, our data demonstrate consistent expression of stress-response genes and production of related metabolites in microbial communities from plant litter in response to a history of drought. The most discernible physiological adaptation to drought was greater abundance of organic osmolytes that function to equilibrate osmotic potential between cells and their surroundings. We found some evidence that the flow of inorganic ions across the cell membrane is also regulated to maintain osmolarity. Gene expression analysis of in situ communities suggests that various compatible solutes like ectoine, betaine, choline and trehalose were employed as osmolytes [6, 10, 31]. Corroborating this inference, under reduced precipitation, we observed significantly higher concentrations of ectoine in grass litter communities and higher concentrations of choline and betaine in shrub litter communities. Another significant physiological signature of drought was synthesis of capsule and EPS that enable cells to form biofilms and retain water for longer periods in drier environments [6, 9, 42]. We also observed a significant increase in expression of genes linked to dormancy and sporulation in the grass reduced precipitation treatment, however only 3 out of 60 genes were identified as indicators of reduced precipitation treatment. These physiological changes likely reflect selection for microbial taxa with traits that confer a fitness advantage under drought.

In ambient precipitation treatments, we observed more growth-related indicators which implies that under less stressful conditions, microbial communities divert fewer resources into stress tolerance and therefore grow better [5, 43]. Our data demonstrate that physiological response to drought trades off against growth by diverting investment away from central metabolism and associated assimilatory pathways such as amino acid and nucleotide synthesis. Physiological adaptation mechanisms like osmolyte accumulation can be energetically very expensive—by some accounts osmotic stress has been estimated to reduce growth yields by nearly 90% [5, 44]. Such shifts in resource allocation can have big impacts on ecosystem C and N balance. To this end, increased stress investment in grass litter communities at the expense of growth may explain decreased decomposition under drought previously observed in the same field experiment [7].

Although we observed a trade off between drought stress tolerance and growth traits in grass communities, shrub litter chemistry appears to alter these community-level trade offs. Compared with grass, shrub leaf litter hosts a taxonomically and functionally more diverse decomposer community. Despite this diversity, it is likely that lower chemical quality (higher *C*:*N* ratio, higher proportion of lignin and other recalcitrant compounds and lower proportion of cellulose, hemicellulose and cell solubles than the grass litter [17]) makes shrub litter harder for microorganisms to degrade and imposes constraints on microbial physiology that affect stress tolerance strategies under drought. We also observed a high frequency and diversity of functional indicators of substrate degradation, uptake, and assimilation in shrub litter communities that may reflect the low resource quality. This high level of investment in resource acquisition traits in shrub litter communities may have affected their response to drought stress if there are trade offs between resource acquisition and stress tolerance traits. Although such trade offs require further validation, they align with a recently-proposed theoretical framework that links ecosystem functioning with omics-derived traits of in situ microbial communities [16].

We aimed to assess the metabolite production of microbial communities in situ, but we cannot rule out that some persistent metabolites were produced by plants (e.g. phenolic compounds such as caffeic acid or nicotinic acid) or by mesofauna living on litter (e.g. carnitine or creatine). Without more work on in situ metabolomics, it is difficult to separate microbial metabolites from other sources. Still, the fast turnover of many monomeric metabolites and their correspondence with microbial gene expression gives us confidence that microbes produced some of the metabolites we discussed. Going forward, these metabolites could be targeted for more sophisticated analyses of origin and fate.

To conclude, 10 years of drought have altered microbial community composition and physiology. Our results indicate that the metabolic costs of microbial stress tolerance to drought trade off against growth traits in drought-selected communities. In addition, litter of poor chemical quality constrains gene expression and metabolite production associated with drought stress tolerance. Substrate quality and investment in resource acquisition traits can thereby alter the stress tolerance-growth trait relationship in microbial communities. The potential linkages of microbial traits with litter decomposition rates suggest that community-level trait trade offs have consequences for organic matter decomposition, a key ecosystem process. Our data clearly identifies some of the physiological mechanisms of community-level adaptations to long-term drought and possible trade offs in stress tolerance, resource acquisition and growth.

## Supplementary information

Supplemental Material

Dataset S1

Dataset S1M

Dataset S2

Dataset S3

Dataset S4

## Data Availability

The authors declare that the data supporting the findings of this study are available within the article and its Supplementary Information file, and from the corresponding author on request. The transcriptomics data discussed in this publication have been deposited in NCBI’s Gene Expression Omnibus [45] and are accessible through GEO Series accession number GSE148618 (https://www.ncbi.nlm.nih.gov/geo/query/acc.cgi?acc=GSE148618).
