# Supplementary material for: Drought and plant litter chemistry alter microbial gene expression and metabolite production
Source: ISME J. 2020 May 22;14(9):2236–47. doi: 10.1038/s41396-020-0683-6 (PMC7608424; doi:10.1038/s41396-020-0683-6)
Supplement: Supplementary file 1 — Supplemental Material [file 41396_2020_683_MOESM1_ESM.pdf]

Supplementary Information

Drought and plant litter chemistry alter microbial gene expression and metabolite production

Malik et al.

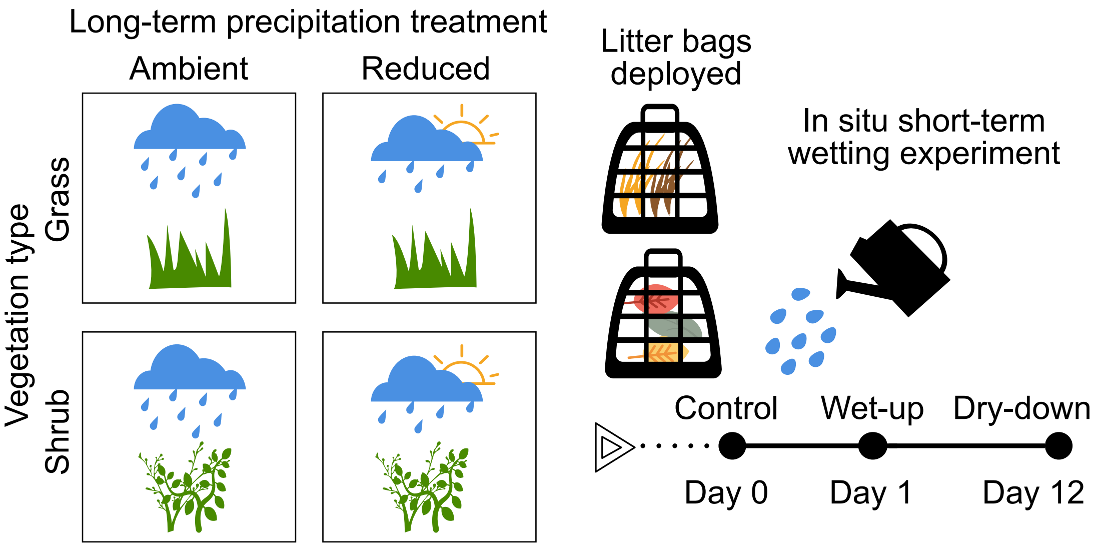

Figure S1: *In situ* wetting was performed on litter bags deployed at the Loma Ridge Global Change Experiment that involved field scale precipitation manipulation (ambient and reduced) for a decade under two vegetation types (grass and shrub). Litter bags (n=4) were harvested before (Sampling 1), one day after (Sampling 2), and 12 days after (Sampling 3) a discrete wetting event.

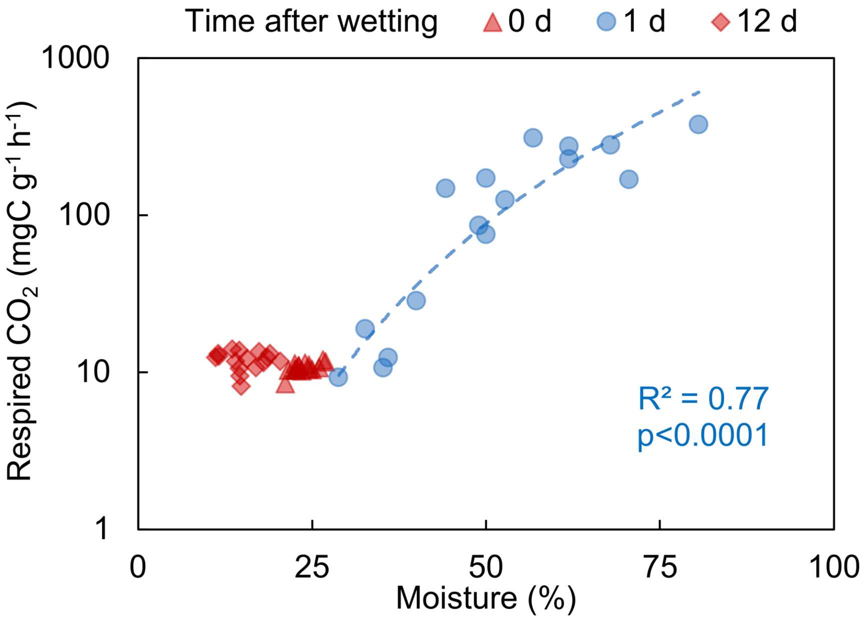

Figure S2: Rate of respired CO_2 in control (day 0, Sampling 1), wet-up (day 1, Sampling 2) and dry-down (day 12, Sampling 3) treatments. Best fitting regression line for the moisture-rate of respired CO_2 relationship: $y = 1E\text{-}05x^{4.04}$. There was a large variation in moisture and respiration rates among replicates within each treatment which meant that the *in situ* wet-up experiment was not ideal to investigate wetting induced shifts in gene expression and metabolite production.

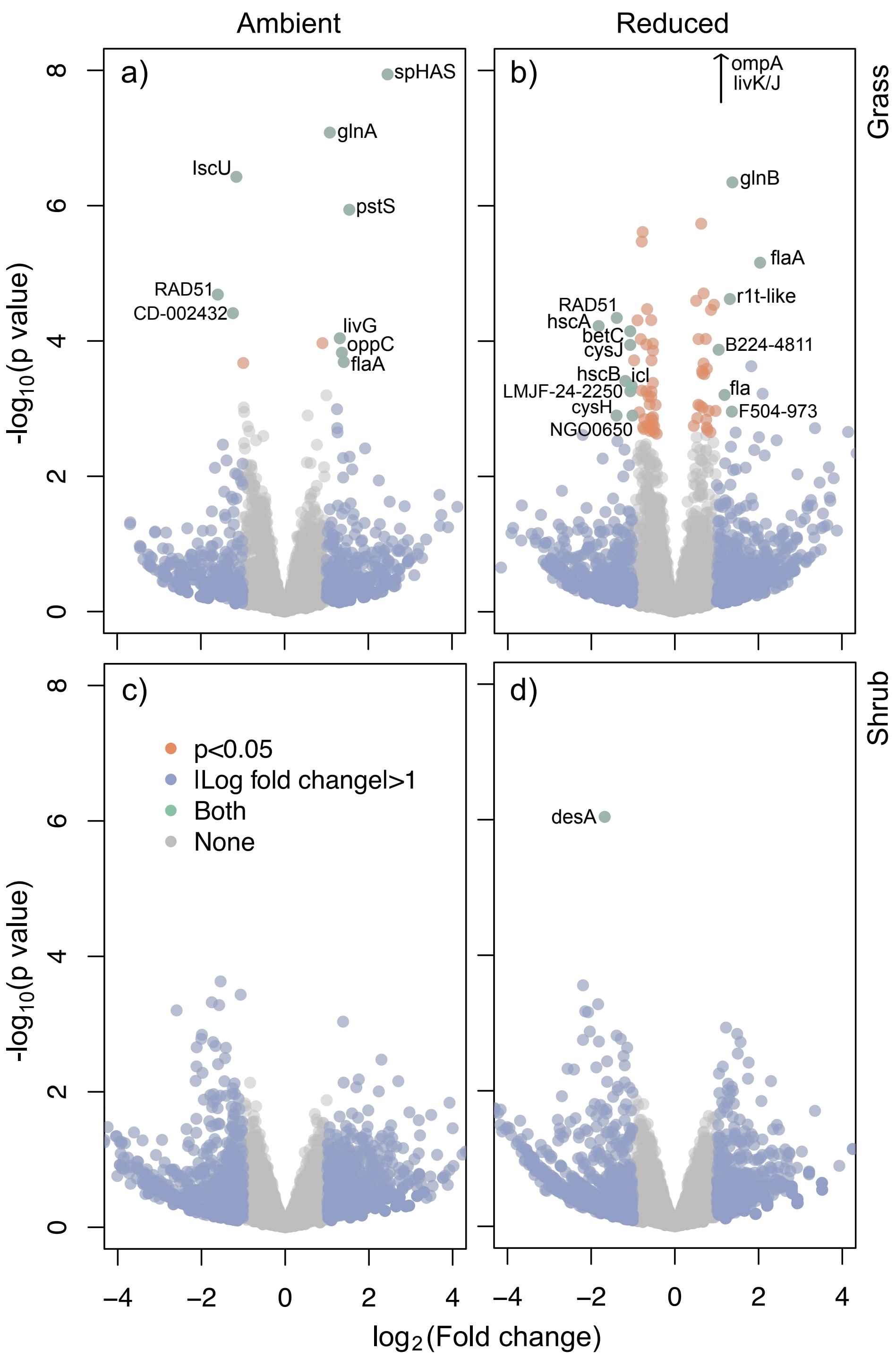

Figure S3: Gene expression patterns in response to *in situ* wetting: Volcano plots of control (day 0) vs. wet-up (day 1) litter showing transcript level changes in (a) grass ambient, (b) grass reduced, (c) shrub ambient and (d) shrub reduced precipitation treatments. Only genes with significant shifts in expression on wet-up with fold change more than double or less than half the control samples were labelled. Relevant upregulated genes- ompA: outer membrane protein A; livG, livK/J: branched-chain amino acid ABC transporter; pstS: phosphate ABC transporter; flaA, fla: flagellin protein; oppC: oligopeptide transport system. Relevant downregulated genes- IscU: iron-sulfur cluster assembly enzyme; rad51: DNA repair protein; hscA, hscB: chaperone protein. More details and discussion can be found in Malik et al. 2019, BioRxiv 631077; doi: https://doi.org/10.1101/631077

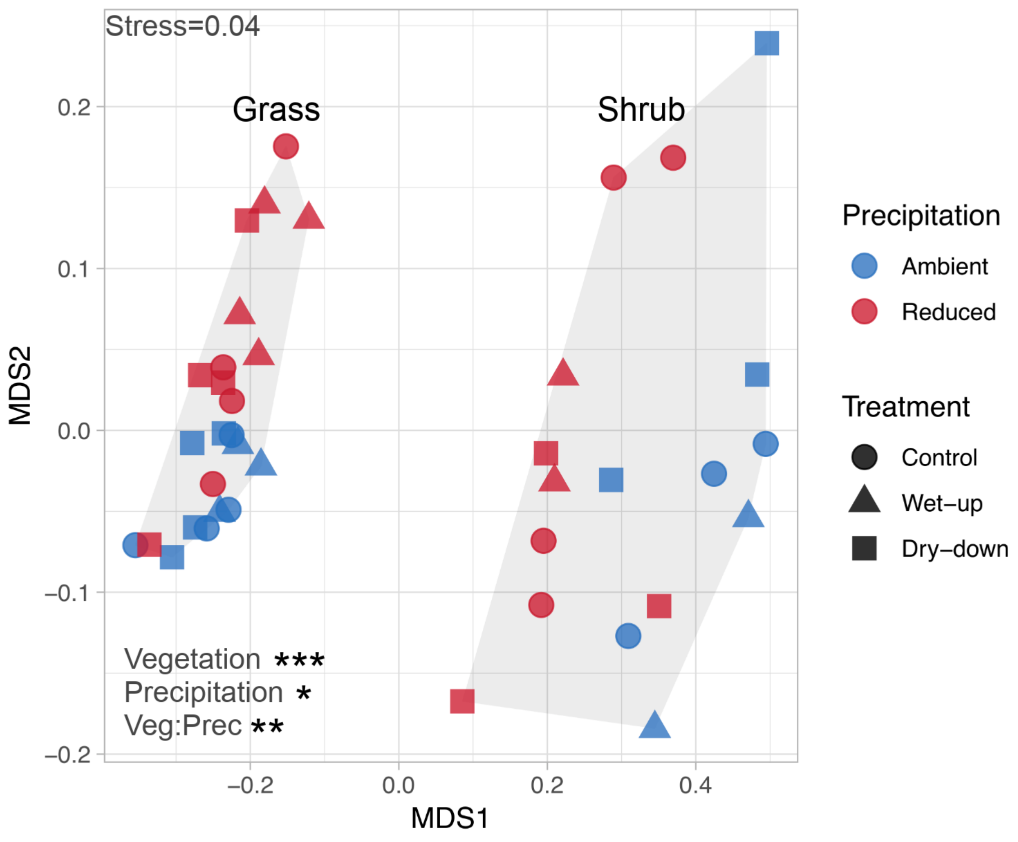

Figure S4: Two dimensional NMDS ordination of communities identified at the genus level from metatranscriptomics based on taxonomic annotations of functional genes (Sampling 1: control, Sampling 2: wet-up, Sampling 3: dry-down). Asterisks mark the significance of treatments that cause clustering of similar samples based on Bray-Curtis dissimilarity index analysed using permutational multivariate analysis of variance (PERMANOVA); *** $p < 0.001$, ** $p < 0.01$, * $p < 0.05$.

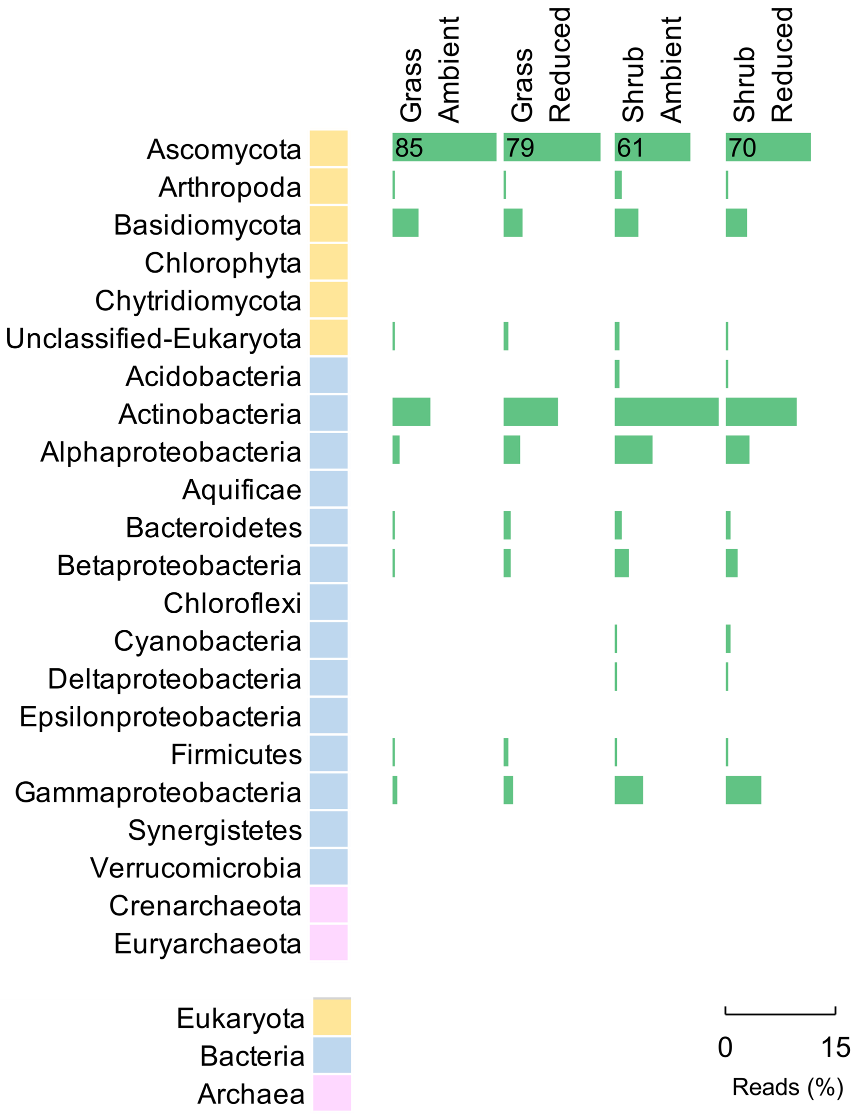

Figure S5: The mean proportion of various phyla/sub-phyla in communities from the litter types and precipitation treatments. Ascomycota was the most abundant phylum, it was disproportionately higher than the rest of the phyla and therefore has been plotted on a separate scale with its abundance labelled on the bar.

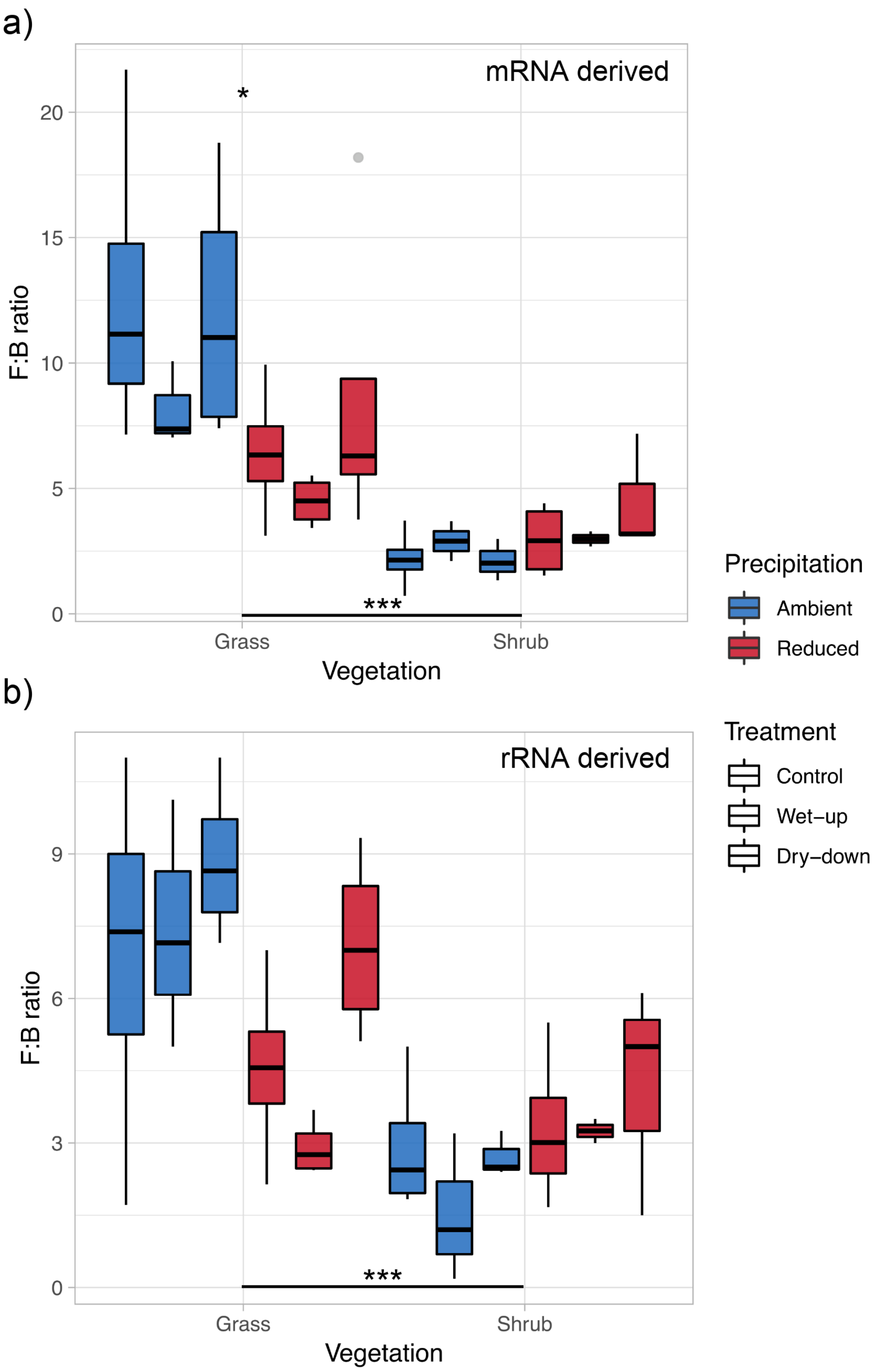

Figure S6: Community fungal:bacterial (F:B) ratio estimated as (a) the ratio of mRNA sequences assigned to fungi and bacteria (b) the ratio of 18S and 16S rRNA abundances in total RNA extracts; n=3-4 (Sampling 1: control, Sampling 2: wet-up, Sampling 3: dry-down). Most of the mRNA (~ 97%) was annotated to fungi or bacteria, therefore we assumed that majority of 16S belonged to bacteria and 18S to fungi. Asterisks mark the significance of differences between groups as analysed by Tukey's multiple comparison test (*** $p < 0.001$, ** $p < 0.01$, * $p < 0.05$).

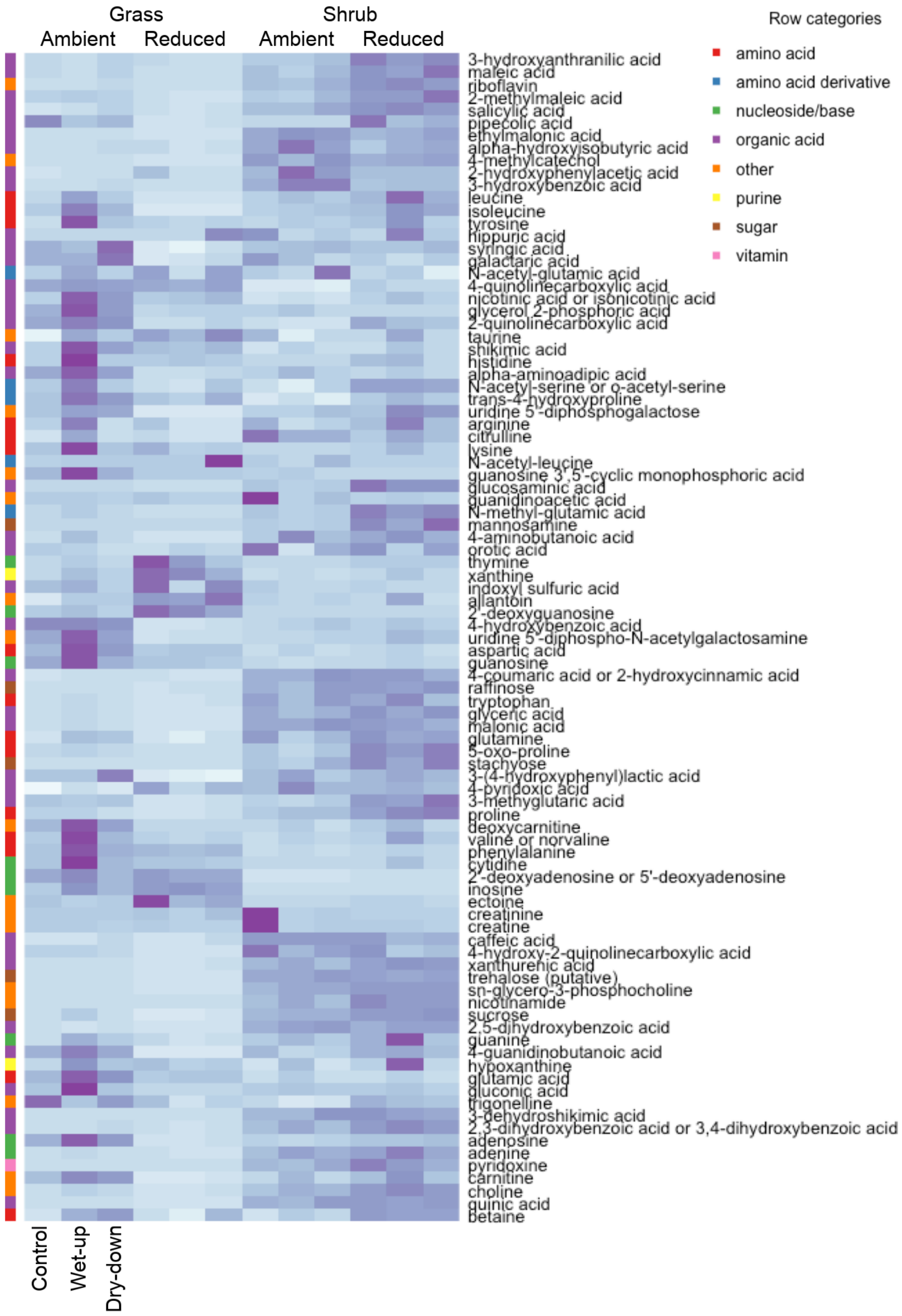

Figure S7: Heatmap of mean peak heights (n=3-4) of all identified metabolites across treatments.

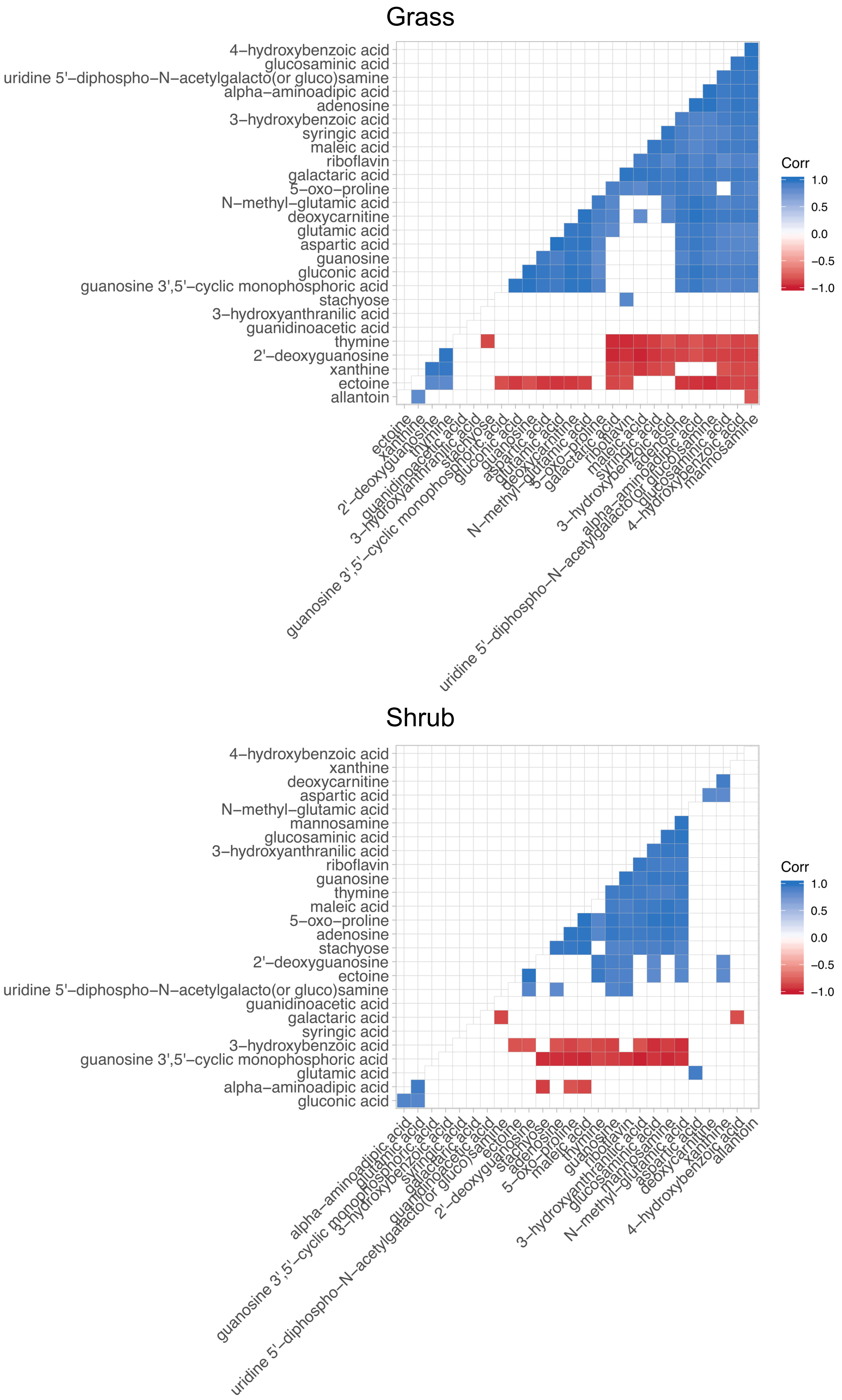

Figure S8: Correlations between metabolites significantly enriched in either ambient or reduced precipitation treatments across the two vegetation types. Empty boxes signify that the correlations are not significant.
